# Antimicrobial effect of probiotic bacteriocins on *Streptococcus mutans* biofilm in a dynamic oral flow chamber model – an *in vitro* study

**DOI:** 10.1080/20002297.2024.2304971

**Published:** 2024-01-28

**Authors:** Elisabeth Reichardt, Viktoriya Shyp, Lea Alig, Carlalberta Verna, Eva M. Kulik, Michael M. Bornstein

**Affiliations:** a Department of Pediatric Oral Health and Orthodontics, University Center for Dental Medicine UZB, University of Basel, Basel, Switzerland; b Department Research, University Center for Dental Medicine UZB, University of Basel, Basel, Switzerland; c Department of Oral Health & Medicine, University Center for Dental Medicine UZB, University of Basel, Basel, Switzerland

**Keywords:** Probiotics, biofilm, antibacterial therapy, Streptococcus salivarius K12, bacteriocin-like inhibitory substances, Streptococcus salivarius M18, Streptococcus mutans

## Abstract

**Aim:**

To determine the antimicrobial activity of the bacteriocin-producing probiotic strains *Streptococcus salivarius* K12 and *Streptococcus salivarius* M18 alone or in combination against caries-associated *Streptococcus mutans*.

**Methods:**

Antimicrobial activity of *S. salivarius* K12 and/or *S. salivarius* M18 against *S. mutans* ATCC 25175 growth and biofilm formation on hydroxyapatite (HA) discs was determined in a flow chamber model by recording the colony forming units (CFU/ml) after 48 h of co-cultivation. The biofilm was analyzed by scanning electron microscopy (SEM) and by confocal laser scanning microscopy (CLSM). Additionally, the simultaneous antagonism assay was used to assess the inhibitory effect of *S. salivarius* K12 and/or *S. salivarius* M18 against *S. mutans* ATCC 25175 and 21 clinical isolates of *S. mutans*.

**Results:**

Co-cultivation of *S. mutans* and *S. salivarius* K12 and/or *S. salivarius* M18 led to the inhibition of *S. mutans* viability, thereby, preventing its biofilm formation on HA discs. Furthermore, *S. salivarius* K12 and *S. salivarius* M18 exhibited antimicrobial activity against most clinical isolates of *S. mutans*.

**Conclusion:**

The *in vitro* flow chamber system used in this study allows the simulation of time-dependent administration of *S. salivarius* probiotic strains, either alone or in combination, to investigate the prevention of *S. mutans* biofilm formation in a standardized model.

## Introduction

The oral cavity is inhabited by complex microbial communities that can form biofilms which may subsequently lead to caries and periodontitis [1]. Caries remains a major public health problem and is characterized by a multifactorial etiology. Under favorable conditions, the cariogenic oral microbiota in the biofilm metabolize dietary carbohydrates resulting in prolonged periods of local demineralization of the enamel, i.e. white spot lesions or cavitated caries of the dentin [2]. Various models exist to explain the development of cariogenic biofilms as well as important influencing factors such as genetic predisposition and the individual composition of the oral microbiome [2]. The formation of a cariogenic biofilm is described by a shift from dynamic stability with early colonizers to a dysbiotic state characterized by a multispecies cariogenic community including mutans streptococci, *Lactobacillus* species, bifidobacteria and opportunistic *Actinomyces* species [2].

Orthodontic fixed appliances promote the accumulation of plaque around brackets and wires. Particularly, predilection sites with an increased risk for gingivitis, demineralization of the enamel, and the proliferation of pathogenic species i.e. *S. mutans* are found between brackets and gingiva [3–5]. Specialized orthodontic brushes, and pharmaceutical products such as antimicrobial mouth rinses and toothpastes appears to be not sufficient to prevent the buildup of pathogenic biofilms [6,7]. Therefore, additional therapeutic measures might be necessary to maintain a good oral hygiene during orthodontic treatment.

One promising treatment strategy includes the use of beneficial bacteria. *S salivarius* K12 and *S. salivarius* M18 are commercially produced probiotics available as single strain lozenges or as strain mixtures. Both strains are producing bacteriocin-like inhibitory substances (BLIS), called salivaricins [8,9]. Salivaricins belong to the large group of lantibiotics, ribosomally synthesized antimicrobial peptides containing the amino acid lanthionine, which show an antibiotic effect against specific bacterial strains [10,11]. However, there is no consensus on how salivaricins affect dental health in detail [12]. Moreover, although a product containing both probiotic strains is commercially available, the antimicrobial effect of *S. salivarius* strains combination has not yet been investigated in detail [13]. The goal of this study is to determine the antibacterial effect of *S. salivarius* K12 and *S. salivarius* M18 alone or in combination on *S. mutans* biofilm development in a dynamic model mimicking real-life clinical biofilm formation on hydroxyapatite discs and metal brackets. The hypothesis of the present study is that the single administration of *S. salivarius* K12 and/or *S. salivarius* M18 inhibits *S. mutans* under these conditions.

## Materials and methods

### Bacterial strains and growth conditions

*S*. *salivarius* K12 (Burgerstein Biotics-O, Fa. Burgerstein, Switzerland) and *S*. *salivarius* M18 Throat Guard® (BLIS Technologies Limited, Wellington, New Zealand) from lozenges were suspended in 3 ml THB (Todd Hewitt Broth, BD, Allschwil, Switzerland) for 1.5 h under aerobic conditions at 37°C. *S. mutans* ATCC 25175, 21 clinical *S. mutans* isolates, *Bacteroides fragilis* ATCC 25285, *Micrococcus luteus* ATCC 6575, *M. luteus* ATCC 6592 and *S. salivarius* MU (a non salivaricin-producer, kindly provided by Prof. J. Tagg, Department of Microbiology and Immunology, University of Otago, Dunedin, New Zealand) were grown on Columbia blood agar plates (BBL^TM^, Becton Dickinson, Allschwil, Switzerland) supplemented with 0.1% calcium carbonate (CaCO_3_) and 5% human blood (Blood donor center, Basel, Switzerland) under anaerobic conditions (80% N_2_, 10% H_2_ and 10% CO_2_) at 37°C for either 24 h or 48 h.

### Anaerobic flow chamber model

The *in vitro* flow chamber model used in this study has been previously described in detail [14]. Briefly, a flow chamber was connected to the bacterial culture dispenser and the peristaltic pump by polyvinylchloride tubes. Sterile hydroxyapatite discs with a diameter of 12.1 mm and a thickness of 2.1 to 2.4 mm (ENAMEL LIKE HA discs, Fa. Himed N.Y.) as substrate for biofilm formation were placed in the flow chamber together with artificial saliva (AS) to simulate human oral conditions [15]. The flow rate was set at 0.8 ml/min to simulate physiological low shear conditions found in oral cavity. The system was incubated under anaerobic conditions (80% N_2_, 10% H_2_ and 10% CO_2_) at 37°C for 48 h on a shaker (260 rev/min) [14]. To mimic the different surfaces (teeth and metal) present during orthodontic treatment, lower incisor brackets (Victory, 3 M Unitek Monrovia, CA) were fixed with dental composite (GC Ortho Connect™, Fa. GC, Tokyo, JPN) on hydroxyapatite discs. Discs without fixed appliances served as control.

**Figure 1.**
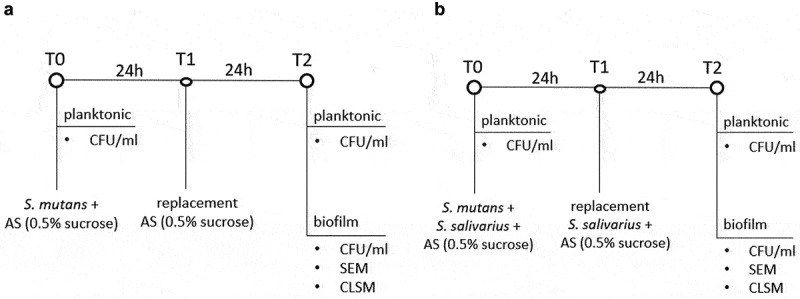
Experimental scheme of the anaerobic incubation of (a) *S. mutans* ATCC 25175 (control) and (b) *S. mutans* and *S. salivarius* co-cultivation in a flow chamber. *S. mutans* ATCC 25175 and *S. salivarius* K12/*S. salivarius* M18 were suspended in 30 ml AS containing 0.5% sucrose (T0). After 24 h (T1), the medium was replaced with 30 ml AS containing 0.5% sucrose (a) or 30 ml AS containing 0.5% sucrose and the probiotic bacteria *S. salivarius* K12 and/or *S. salivarius* M18 (b). Bacteria were incubated for additional 24 h (T2). Planktonic bacteria were quantified at the beginning of the experiment (T0) and after 48 h of incubation (T2). The biofilm formed on the HA discs after 48 h (T2) was analyzed by determining the CFU/ml, SEM, and CLSM.

A co-cultivation model in the anaerobic flow chamber was implemented to assess the antimicrobial activity of *S. salivarius* K12 and/or *S. salivarius* M18 on *S. mutans* in a biofilm (Figure 1). For this, 10 single colonies of *S. mutans* grown on Columbia blood agar plates (CBA) were transferred to 30 ml THB containing 0.5% sucrose. After 24 h of anaerobic incubation at 37°C, 3 ml of suspension was diluted in 27 ml of 0.9% NaCl. *S. salivarius* K12 resp. *S. salivarius* M18 were extracted from lozenges as described above and 100 µl of the respective suspensions were plated on CBA and incubated anaerobically at 37°C. After 24 h, single colonies of either *S. salivarius* K12 or *S. salivarius* M18 were resuspended in 30 ml 0.9% NaCl, ultrasonicated for 30 s (22.5 W, Vibracell, Sonics & Materials, Newtown, CT) and centrifuged at 9200 rpm for 5 min at room temperature. The bacterial pellets were resuspended in 30 ml AS containing 0.5% sucrose and incubated in the flow chamber with one direction circulation mimicking oral saliva flow. Free-floating bacteria present in the tubing and dispenser represented the planktonic population, while bacteria attached to the HA discs were considered as biofilm-associated.

For the co-cultivation, *S. mutans* ATCC 25175 was mixed with a single strain of *S. salivarius* K12 or *S. salivarius* M18 or with *S. salivarius* K12 and *S. salivarius* M18 combination in 1:1 or 1:1:1 ratio, respectively (T0). The biofilms were then allowed to form on the HA discs by placing them for 48 h in the flow chamber. The incubation time of 48 h was chosen to enable one medium exchange and re-administration of the probiotic strains. Therefore, after 24 h (T1), the medium was replaced with 30 ml AS supplemented with 0.5% sucrose and *S. salivarius* K12 and/or *S. salivarius* M18. After additional incubation for 24 h, the flow chamber system was stopped and the planktonic and the biofilm-associated bacteria were analyzed (T2). A flow chamber system with *S. mutans* only was used as a positive control (Figure 1).

### Quantification of bacteria

To quantify the number of viable bacteria present in the biofilm formed on the HA discs, the discs were removed from the flow chamber system, dipped gently in 0.9% NaCl to remove any non-adherent cells and vortexed in 5 ml 0.9% NaCl for 1 min. For each suspension, serial dilutions were made in 0.9% NaCl and 100 µl of appropriate dilutions were plated on Mitis Salivarius Agar (Difco™ Mitis Salivarius Agar, BD, Allschwil, Switzerland), a medium used for the selective isolation of Streptococcal species [16]. To determine the number of viable bacteria in the planktonic suspensions, serial dilutions were made in 0.9% NaCl and 100 µl of appropriate dilutions were plated on Mitis Salivarius Agar.

Planktonic and biofilm-associated bacteria were quantified at the start of the experiment (T0) and after 48 h of incubation (T2) (Figure 1).

### Fluorescence in situ hybridization (FISH) and CLSM

High-pressure liquid chromatography (HPLC)-purified oligonucleotide probes for *Streptococcus spp*. and *S. mutans* were commercially synthesized and 5’-end modified with Cy3 and Cy5 fluorophores, respectively (Microsynth, Balgach, Switzerland) (Table 1). Appropriate probe sequences for the specific detection of each bacterial strain in the biofilm have been described previously [17]. Three biofilm-coated discs with *S. salivarius* K12 resp. *S. salivarius* M18 and *S. mutans* ATCC 25175 biofilms were fixed in 4% paraformaldehyde in physiological saline (NaCl 0.9%) for 1 h at 4°C and washed twice with 0.9% NaCl with RNase inhibitor (Guard® RNase Inhibitor, Sigma-Aldrich, Switzerland). Thereafter, the samples were permeabilized by exposure to 7 mg/ml of lysozyme (Fluka, Buchs, Switzerland) in lysis buffer (0.1 M Tris-HCl [pH 7.5], 5 mM EDTA) for 10 min at 37°C in a humid chamber, and rinsed with physiological saline with RNase inhibitor. Non-specific hybridization was blocked with Denhardt’s solution for 1 h at 37°C. For hybridization, HA discs were placed in 96-well polystyrene plates and incubated in hybridization buffer (0.9 M NaCl, 20 mM Tris-HCl [pH 7.5], 25% (vol/vol) formamide, and 0.01% (wt/vol) sodium dodecyl sulfate) containing streptococci oligonucleotide probes (10 ng/ul) at 46°C for 3 h. After hybridization, the biofilms were immediately transferred into washing buffer (102 mM NaCl, 20 mM Tris-HCl [pH 7.5], 5 mM EDTA, 0.01% SDS) at 48°C for 30 min. Biofilms were examined using a Leica SP8® microscope (Leica, Wetzlar, Germany). The images were collected using 520–600 nm and 650–730 nm emission spectrum for Cy3 and Cy5, respectively. Confocal images were obtained using a ×63 oil immersion objective. Each biofilm was scanned at three arbitrary positions at the center of the disc. Z-direction series were generated by vertical optical sectioning at every position with the thickness of the slices set to 0.46 µm.

**Table 1.** Characteristics of 16S rRNA gene-directed oligonucleotide probes used for FISH.

Probe name	Tag	Target	Oligonucleotide sequence (5`-> 3`)	Reference
MUT590	Cy3	*Streptococcus mutans*	ACT CCA GAC TTT CCT GAC	[17]
STR405	Cy5	All streptococci	TAG CCG TCC CTT TCT GGT	[17]

### Scanning electron microscopy (SEM)

Biofilm-coated HA discs with or without brackets were used for SEM. Bacteria were fixed overnight in 2% glutaraldehyde solution (Sigma, Buchs, Switzerland), washed once with phosphate buffered saline (PBS, ThermoFisher, Waltham, MA), and dehydrated in stepwise increasing concentrations of ethanol: 30%, 50%, 70%, 90% and 100% for 10 min each. The samples were then critical-point-dried and coated with 10 nm of gold and examined by scanning electron microscopy (Fei Nova NanoSEM 230®, Eindhoven, the Netherlands) [18].

### Antimicrobial activity of *S. salivarius* K12 and/or *S. salivarius* M18 - simultaneous antagonism method

Antimicrobial activity of *S. salivarius* K12 and *S. salivarius* M18 was determined using the simultaneous antagonism method as previously described [9]. Briefly, overnight cultures of the respective indicator strains were prepared in 3 ml Todd-Hewitt Broth (THB; BD, Allschwil, Switzerland), adjusted to a turbidity equivalent of a McFarland 0.5 standard and 100 µl of the respective suspensions were plated on CaCO_3_ (CBA) plates. Single colonies of *S. salivarius* K12 and/or *S. salivarius* M18, respectively, were picked and stabbed successively into each agar plate. The plates were subsequently incubated at 37°C for 48 h under anaerobic conditions. After incubation, the zones of inhibition surrounding the *S. salivarius* colonies were measured: No inhibition 0 mm (−); Slight inhibition 1–2.5 mm (+); Inhibition 2.5–4 mm (++); Strong inhibition 4–11.0 mm (+++).

### Data analysis and statistics

All experiments were performed at least in biological triplicates. Data were analyzed using a two-way ANOVA, calculated and visualized with GraphPad Prism (Version 9.0.0 for Windows, GraphPad Software, San Diego, California, USA). Differences in data points were considered significant at *p* < 0.05 (*), *p* < 0.01 (**), *p* < 0.001 (***).

## Results

### *S. mutans* biofilm formation on oral surfaces in the flow chamber

Using an *in vitro* flow chamber system, which mimicked the oral cavity microenvironmental conditions, mature biofilm of *S. mutans* was formed on HA discs with or without orthodontic brackets after 48 h. The structure of the biofilms analyzed by SEM revealed abundant adherent clusters of *S. mutans* on both, HA discs surface and on bonded orthodontic brackets. There was a notable increase in number of *S. mutans* cells on HA discs with brackets compared to HA discs without brackets (Figure S1).

**Figure 2.**
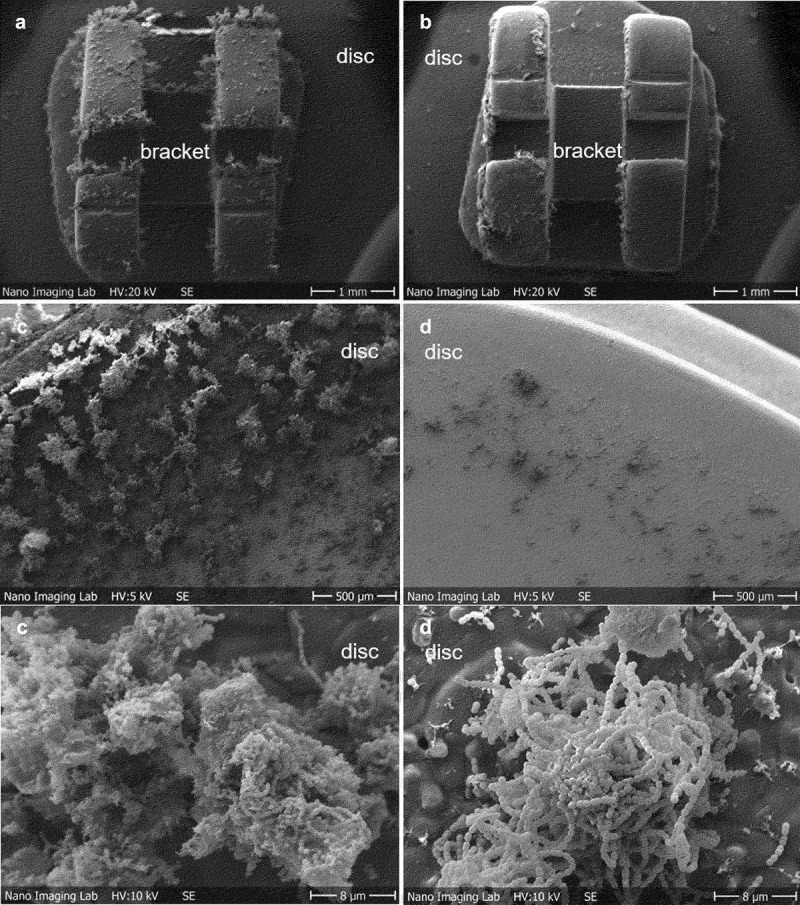
Representative SEM images of the biofilm-coated HA discs after 48 h of anaerobic incubation in the flow chamber. *S. mutans* monoculture biofilm (negative control) formed on HA discs with brackets (a) and without brackets (c). *S. salivarius* M18 and *S. mutans* mixed biofilm formed on HA discs with (b) and without bonded orthodontic brackets (d) after 48 h of co-cultivation. For biofilm on HA discs with brackets, images were taken at x20. For biofilm on HA discs without brackets, images were taken at x50 and x3000.

**Figure 3.**
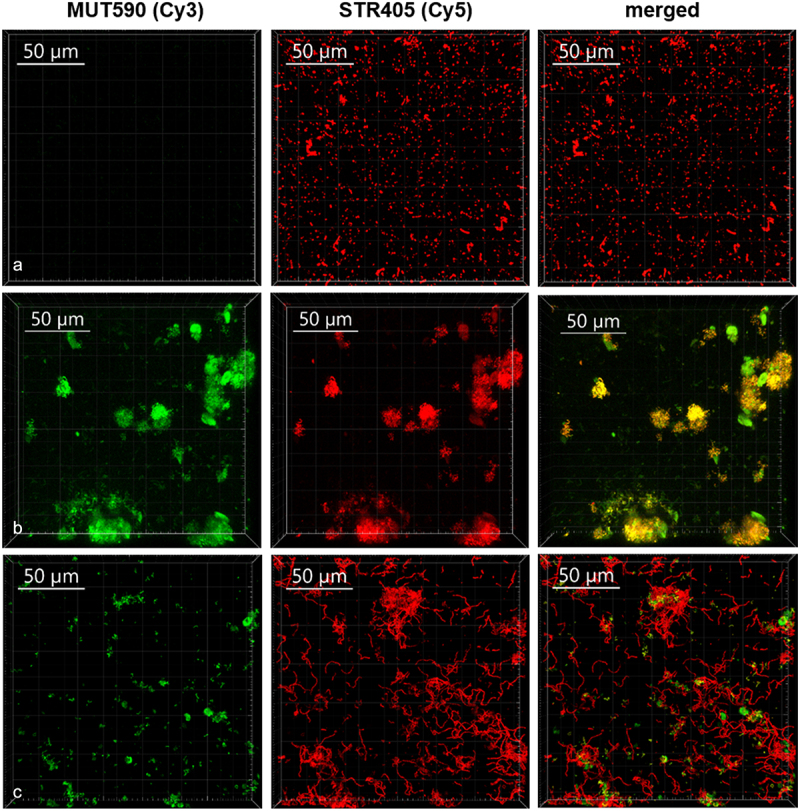
The composition of biofilm formed on HA discs after 48 h of incubation as monitored by FISH. Representative CLSM images of the biofilm formed by *S. salivarius* M18 (a) and *S. mutans* ATCC 25175 (b) monocultures, as well as *S. mutans* and *S. salivarius* M18 mixture after co-cultivation (c). Biofilms were labeled with MUT590 (green) and STR405 (red) probes.

The co-cultivation of *S. mutans* with *S. salivarius* K12 and/or *S. salivarius* M18 significantly reduced the biofilm formation on both surfaces (Figure 2(b)). To display the composition of the biofilm after the co-cultivation, CLSM combined with FISH was used for selective detection of bacteria (Table 1). Thus, pan-reactive probe SRT450 was used to stain all streptococci in the biofilm, while MUT590 probe specifically distinguished *S. mutans* (no signal on *S. salivarius*, Figure 3(a)). CLSM validated further *S. mutans* and *S. salivarius* M18 co-clustering, indicating a higher proportion of the probiotic strain in the co-cultivation biofilm (Figure 3). Analogous results were found when *S. salivarius* K12 was used (data not shown). Interestingly, neither *S. salivarius* K12 nor *S. salivarius* M18 adhered well to HA discs or other material when grown as monoculture. However, both strains formed a solid biofilm in the presence of *S. mutans* (Figures 2 and 3). Additionally, *S. salivarius* formed noticeable longer streptococcal chains after co-cultivation with *S. mutans* (Figure 3).

### Antimicrobial activity of *S. salivarius* K12 and/or *S. salivarius* M18 in a co-cultivation model

**Figure 4.**
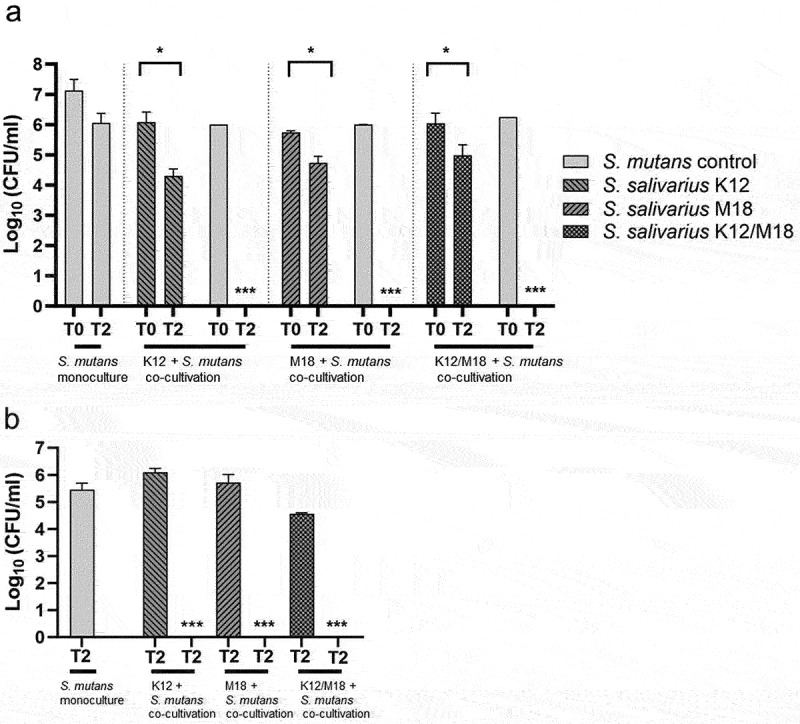
Antimicrobial activity of *S. salivarius* K12 and/or M18 against *S. mutans* in a co-cultivation model. (a) bacterial viability measured as CFU/ml for planktonic cells at the beginning (T0) and after 48 h (T2) of co-cultivation of *S. mutans* ATCC 25175 (light grey column) with *S. salivarius* K12 (grey column, left right diagonal hatch pattern), M18 (grey column, right left diagonal hatch pattern) resp. and K12/M18 mixture (dark grey column, crossed line hatch pattern). *S. mutans* monoculture was used as a control (light grey column). (b) bacterial viability measured as CFU/ml for biofilm-associated cells after 48 h (T2) of co-cultivation of *S. mutans* (light grey column) with *S. salivarius* K12 (grey column, left right diagonal hatch pattern), M18 (grey column, right left diagonal hatch pattern) and resp. K12/M18 (dark grey column, crossed line hatch pattern). *S. mutans* ATCC 25175 monoculture 48 h biofilm was used as a control (light grey column).

The co-cultivation of *S. mutans* ATCC 25175 with *S. salivarius* K12 and/or *S. salivarius* M18 led to a complete inhibition of *S. mutans* viability in the planktonic suspension, i.e. the bacterial suspension present in the tubing of the flow chamber system after 48 h. In contrast, the cell numbers of either *S. mutans* grown as monoculture or the probiotic strains *S. salivarius* K12 and *S. salivarius* M18 when grown in the co-culture together with *S. mutans*, remained high but notably decreased over time (Figure 4(a)), presumably, because both were retained in the growing biofilms. Consequently, no viable *S. mutans* could be detected in the biofilm on HA discs after 48 h of co-cultivation (Figure 4(b)).

### Antimicrobial activity of *S. salivarius* K12 and/or *S. salivarius* M18 - simultaneous antagonism method

Using a simultaneous antagonism assay, *S. salivarius* K12 and/or *S. salivarius* M18 exhibited antimicrobial activity against most of *S. mutans* isolates tested (Table 2). However, differences in sensitivity were noticed. Thus, *S. mutans* ZIB6095 was not inhibited by *S. salivarius* M18, but was sensitive against *S. salivarius* K12. Additionally, *S. salivarius* K12 and/or *S. salivarius* M18 were able to inhibit the known indicator strains *M. luteus* 6575, *M. luteus* 6592, and *S. salivarius* MU. When *S. salivarius* K12 and *S. salivarius* M18 were used simultaneously, no significant additive effects were found. Additionally, *S. salivarius* strains K12 and *S. salivarius* M18 inhibited each other (Table 2).

**Table 2.** Inhibition of bacterial growth by *S. salivarius* K12 and M18 as determined by the simultaneous antagonism test. (−) No inhibition; (+) slight inhibition (1–2.5 mm); (++) inhibition (2.5–4 mm); (+++) strong inhibition (4–11.0 mm).

Indicator strains	*S. salivarius* producer strain K12	*S. salivarius* producer strain M18	*S. salivarius* producer strains K12/M18
*S. mutans* ZIB6111	++	++	++
*S. mutans* ZIB6113	++	+	++
*S. mutans* ZIB6117	+++	+++	+++
*S. mutans* ZIB6118	+	++	++
*S. mutans* ZIB6119	++	++	++
*S. mutans* ZIB6120	++	++	++
*S. mutans* ZIB6121	++	+	++
*S. mutans* ZIB6123	++	++	++
*S. mutans* ZIB6551	++	+	++
*S. mutans* ZIB6583	++	++	++
*S. mutans* ZIB6086	++	++	++
*S. mutans* ZIB6087	++	++	++
*S. mutans* ZIB6088	++	++	++
*S. mutans* ZIB6090	++	++	++
*S. mutans* ZIB6091	++	++	++
*S. mutans* ZIB6095	++	-	++
*S. mutans* ZIB6092	++	++	++
*S. mutans* ZIB6099	++	++	++
*S. mutans* ZIB6101	++	++	++
*S. mutans* ZIB6107	++	+	++
*S. mutans* ZIB6109	++	++	++
*M. luteus* 6575	+++	+++	+++
*M. luteus* 6592	+++	+++	+++
*S. mutans* ATCC 25175	++	++	++
*S. salivarius* MU (non salivaricin-producer)	++	++	++
*S. salivarius* K12	-	++	++
*S. salivarius* M18	++	-	++
*B. fragilis* ATCC 25285	-	-	-

## Discussion

Despite being a preventable disease, dental caries remains a major public health problem for both children and adults [19]. Especially, fixed orthodontic therapy enhances the risk for oral diseases due to increased plaque accumulation and white spot lesions [3,5–7]. Recently, probiotic approaches using *S. salivarius* lozenges have been developed to modulate the bacterial community in dental plaque by administering beneficial organisms that belong to the natural healthy plaque community [10].

Approximately 700 bacterial species are known to be part of the oral microbiome. In the oral cavity, bacteria find various sites for attachment, especially on hard tissues such as teeth, where they can accumulate to form biofilms [20]. They may coexist with their host; however, dysbiosis may occur, leading to oral diseases such as caries or periodontitis. *Streptococcus* species belong to the first colonizers of the oral cavity and are also ubiquitously present, making them the predominant genus in the oral cavity [21]. *S. salivarius* belongs to the commensal oral flora and may even be beneficial to the host by producing molecules that inhibit pathogenic species. *S. mutans* is considered a cariogenic bacterium due to its ability to acidify the oral environment. In contrast, certain streptococcal species, such as *S. gordonii* or *S. salivarius*, are known to produce large amounts of alkali or generate hydrogen peroxide that can inhibit the growth of *S. mutans* [21].

*S. salivarius*, in particular strains *S. salivarius* K12 and *S. salivarius* M18, are of interest as they offer numerous potential benefits for the host such as prevention and treatment of pharyngitis, caries and halitosis [4,22–24]. However, to date, the effect of *S. salivarius* isolates *S. salivarius* K12 and *S. salivarius* M18 on dental plaque formation reported in controlled clinical trials remains controversial [4,25]. Moreover, no standardized *in vitro* model, that would allow the analysis of different conditions, is available yet. Such a model could assist in analyzing the effect of individual *S. salivarius* probiotics or their use in combination and facilitate the comparison of these *in vitro* results with the varying results of clinical studies. In a randomized double-blind, placebo-controlled trial, Burton et al. reported that regular consumption of *S. salivarius* M18 for 3 months reduced dental plaque scores in children [26]. Similarly, a significant decrease in plaque accumulation was reported in a pilot randomized clinical trial after four weeks of daily consumption of *S. salivarius* K12 lozenges within 31 patients [27]. In contrast, a prospective, randomized, triple-blind, placebo-controlled trial with 64 patients undergoing fixed orthodontic treatment revealed that daily administration of *S. salivarius* M18 reduced the level of halitosis, but had minimal effects on plaque index (PI), gingival index (GI), and dental biofilm microflora [4]. The reported differences might be attributed to the different treatment regimes, cultivation mode, or probiotics concentration. Therefore, the establishment of an adjustable *in vitro* model mimicking the dynamic condition found in the oral cavity is essential for investigating the effect of probiotics administration on dental biofilm formation. The dynamic flow chamber system used in this study allows the investigation of different parameters of biofilm formation in a low-shear environment with regular medium exchange, flexible cultivation mode, and offers the possibility to employ different oral surfaces with subsequent structural and functional analysis of the biofilms formed.

The cultivation of *S. mutans* in our flow chamber system with fixed orthodontic material showed a significant increase in *S. mutans* biofilm formation especially on orthodontic brackets and HA discs. Potentially, the larger surface area and niches of orthodontic brackets serve as colonization promoters for *S. mutans* biofilm formation on HA discs with bonded brackets in comparison to HA alone. These findings agree with previous observations, where orthodontic materials enhanced the presence of *S. mutans* in patients [3,28].

The reduction of dental plaque and decrease in the viable number of planktonic and biofilm-associated *S. mutans*, as shown in this study, might therefore be an effective measure for caries control. Both, *S. salivarius* K12 and/or *S. salivarius* M18 were able to reduce *S. mutans* biofilm formation on HA discs with or without brackets under anaerobic conditions. Closer examination of bacterial biofilm after co-cultivation revealed that, first, *S. salivarius* K12 and/or *S. salivarius* M18 adsorbed noticeable better on oral surfaces only in the presence of *S. mutans*. Second, in biofilm *S. salivarius* co-clustered with *S. mutans* micro-colonies adhering on top and around these colonies. The term coaggregation was previously described as a clumping phenomenon that occurred when sucrose-grown streptococci were co-cultivated with actinomyces species [20]. Interestingly, the mechanism of coaggregation is essential for the development of complex biofilms and seems to be a prerequisite for antibacterial activities of colonizers such as *S. salivarius*. Our results are in accordance with the view that the structure and/or components of an existing biofilm provide a basis for the inhibition and even elimination of viability of caries-associated bacteria by *S. salivarius*. However, further studies concerning the mechanisms of *S. salivarius* adherence to dental biofilm are necessary.

The administration of a single strain and a combination of *S. salivarius* K12 and *S. salivarius* M18 fully inhibited the viability of *S. mutans* in planktonic culture and, therefore, prevented the formation of viable cariogenic biofilm. The selective antibacterial effect of *S. salivarius* on *S. mutans* including its various clinical isolates was also confirmed in the simultaneous antagonism assay. Notably, while being ineffective against the control strain *B. fragilis, S. salivarius* strains exhibited an antagonistic effect against each other. Moreover, the combination of both strains did not indicate any synergistic effect on *S. mutans* viability, which was claimed by the manufacturers. This might be explained by the diversity of lantibiotics gene profiles encoded by different *S. salivarius* strains [10]. Both, *S. salivarius* K12 and *S. salivarius* M18 are producing multiple bacteriocins, each with its specific antimicrobial activity. *S. salivarius* K12 is known to produce the lantibiotics SalA and SalB, while whole-genome sequencing of *S. salivarius* M18 revealed a novel bacteriocin, salivaricin M, in addition to the already described lantibiotics SalA2 and Sal9 [10]. Although the antibacterial activity of *S. salivarius* is most likely caused by these lantibiotics, its bacteriocin-deficiency variants need to be further tested to dissect the exact mechanism of strong *S. mutans* inhibition. Moreover, *S. salivarius* strains could produce other metabolites and enzymes such as urease with potentially modulatory activity on salivaricins and overall probiotic’s competitiveness [21,24].

Probiotics from oral *S. salivarius* species seem to have no negative impact on the salivary microbiome. Daily administration of *S. salivarius* K12 in a chewing gum did not significantly alter the salivary microbiome and did not increase inflammatory markers in the oral cavity of healthy patients [11]. Furthermore, investigation of *S. salivarius* K12 effect on host epithelial cells revealed that probiotic bacteria downregulated inflammatory response while stimulating anti-inflammatory reactions and adhesive properties of the host cell [11]. This makes *S. salivarius* a prominent probiotic for selective treatment of pathogenic dental biofilms.

While the *in vitro* model system used in this study is designed to simulate dynamic environment conditions during the dental biofilm formation, only a few parameters were tested so far. However, the flow chamber model would allow to further investigate time- and concentration-dependent application of probiotics on biofilm formed on various dental surfaces. Moreover, the effect of probiotics on pre-formed *S. mutans* biofilm is of particular interest for future studies. Finally, to optimize the effect of *S. salivarius* K12 and/or *S. salivarius* M18 application on dental biofilm, multispecies dental biofilm formed on various surface could be also tested with the current model.

## Conclusions

The oral microbiome is a dynamic, complex system comprising multiple bacterial species that contribute to the pathogenic biofilms and subsequently cause caries and gingival inflammation of the surrounding tissues. Despite multispecies etiology, *S. mutans* remains one of the most common and, hence, most important cariogenic pathogen among oral streptococci. Natural antimicrobial compounds produced by oral probiotic streptococci can be utilized as a highly promising treatment option to eliminate *S. mutans*. In the present study, the *in vitro* flow chamber system allows the accurate simulation of daily administration of oral probiotics which was used to show antibacterial effect of *S. salivarius* K12 and *S. salivarius* M18 on *S. mutans* biofilm. Furthermore, our flow chamber system turned out to be especially useful for deciphering the complex dynamic behavior of the oral microbiome under the special caries-promoting conditions during orthodontic treatment.

## Supplementary Material

Bio_Author.docx
