# Supplementary material for: Antimicrobial effect of probiotic bacteriocins on Streptococcus mutans biofilm in a dynamic oral flow chamber model – an in vitro study
Source: J Oral Microbiol. 2024 Jan 28;16(1):2304971. doi: 10.1080/20002297.2024.2304971 (PMC10823884; doi:10.1080/20002297.2024.2304971)
Supplement: Bio_Author.docx [file ZJOM_A_2304971_SM7279.docx]

BIOGRAPHICAL SKETCH

NAME: Elisabeth Caroline Reichardt

POSITION: **Assistant professor,** Doctor of Dental Surgery, Dr. med. dent.

EDUCATION/WORK:

| INSTITUTION | DEGREE | PERIOD | FIELD |
| --- | --- | --- | --- |
| 1. Education   University of Würzburg, Germany  University of Würzburg, Germany  University of Würzburg, Germany  University of Jena, Germany  University of Basel, Switzerland | Bachelor  Master  Dr. med. dent.  Specialist in Orthodontics  Master of advanced studies in lingual orthodontics (MAS) | 10/2005-  10/2007  10/2007-  12/2009  08/2008-  02/2010  04/2015-  08/2017  01/2020-  05/2023 | Dentistry  Dentistry  Microbiology  Orthodontics  Orthodontics |
|  | | | |
| 1. Work   Private Practice  University of Jena, Germany  *Department of Orthodontics*  Charité - University of Berlin, Germany  *Department of Dentofacial Orthopedics, Orthodontics and Pedodontics*  University Center for Dental Medicine UZB Basel, University of Basel, Switzerland  *Department of Pediatric Oral Health and Orthodontics* | Dentist  Specialist in orthodontics,  Postdoctoral researcher  Specialist in orthodontics,  Postdoctoral researcher  **Assistant professor**,  Specialist in orthodontics | 01/2010-  03/2015  04/2015-  08/2017  08/2017-  08/2019  09/2019-  Present | Dentistry  Orthodontics,  Microbiology  Orthodontics and craniofacial  anomalies,  Microbiology  Orthodontics and craniofacial  anomalies,  Microbiology |
